# Bladder Lipoma Associated with Urinary Tract Infection

**DOI:** 10.1100/tsw.2008.91

**Published:** 2008-06-13

**Authors:** Christopher Brown, Adam Jones

**Affiliations:** Royal Berkshire Hospital, Harold Hopkins Department of Urology, Reading, UK

**Keywords:** bladder, lipoma, infection

## Abstract

Lipomas are benign overgrowths of adipose tissue. They are an unusual finding within the bladder wall. There have been five reports[1,2] of bladder lipomas associated with micro- and macroscopic haematuria. We report, to our knowledge, the first bladder lipoma associated with a microbiologically confirmed urinary tract infection.

